# Supplementary material for: Significant enhancement of proton conductivity in solid acid at the monolayer limit
Source: Nat Commun. 2024 Mar 27;15:2706. doi: 10.1038/s41467-024-46911-7 (PMC10973524; doi:10.1038/s41467-024-46911-7)
Supplement: Supplementary file 1 — Supplementary Information [file 41467_2024_46911_MOESM1_ESM.pdf]

Supplementary Information

Significant enhancement of proton conductivity in solid acid at the monolayer limit

Zhang *et al*.

Supplementary Methods

Materials

Hydrochloric acid (HCl, 36.0 – 38.0 wt%), lithium fluoride (LiF, purity ≥98 wt%), sulfuric acid (H_2SO_4, 98 wt%) and hydrogen peroxide (H_2O_2, ≥30 wt%) were purchased from Sinopharm Chemical Reagents (Shanghai) Co., Ltd.. The Ti_3AlC_2 powders (400 mesh) were purchased from Jinlin 11 Technology Co., Ltd.. Polyethylene terephthalate film (PET) (type: A4300, thickness: 125 μm) was purchased from Toyobo Co., Ltd.. Poly(vinyl alcohol) (PVA) (95 wt%, Mw: 89000 – 98000) was purchased from Shanghai Macklin Biochemical Technology Co., Ltd.. The Nafion solution (equivalent weight: 1100, 5 wt% in lower aliphatic alcohol and water) was purchased from Sigma-Aldrich Co., Ltd..

Synthesis of MXene dispersion

The $Ti_3C_2T_x$ MXene nanosheets aqueous dispersion was prepared by the classic LiF + HCl etching process[1]. In detail, Ti_3AlC_2 powder (~1.0 g) was slowly added into the mixture of LiF (~1 g) and HCl (9 M) at 35 °C. Then, the obtained suspended mixture was stirred for 48 h at 40 °C. After the suspended mixture was cooled to room temperature, the collected solid residue was washed with deionized water through centrifugation at 2257 g to obtain colloidal MXene with PH of 6 – 7. Finally, the colloidal MXene was added into 50 mL of deionized water followed by sonication and centrifugation at 1106 g to obtain a stable colloidal suspension of $Ti_3C_2T_x$ MXene nanosheets.

Synthesis of Graphene oxide (GO) dispersion

The GO dispersion was synthesized by the modified Hummer's method[2]. In detail, the graphite flakes (~2 g, lateral size 7 – 10 μm, Alfa Aesar) were first added to concentrated H_2SO_4 (~48 mL) with stirring. $KMnO_4$ (~6 g) was then added slowly into the suspension under ice batch. After keeping the reaction at 35 °C for 4 h, the deionized water (~60 mL) was added slowly until the solution was cooled to room temperature. Additional water (~100 mL) was added followed by a slow addition of H_2O_2 (~2 mL). The resulting graphite oxide was washed with deionized water through centrifugation and exfoliated by sonication. After removing the residues by centrifugation at 13000 g, the GO nanosheets dispersion was obtained with the conductivity <100 μS cm^{-1}.

Fabrication of micro-supercapacitors (MSCs)

The m-$HSbP_2O_8$-MXene MSCs were fabricated by patterning MXene electrodes and subsequent m-$HSbP_2O_8$ dispersion casting. The MXene film was first prepared by vacuum filtration, and then transferred onto the PET substrate under ~5 MPa. After that, the MXene film was patterned to obtain interdigital electrodes by using a laser machining (wavelength: 1060 nm; PL100 Fiber laser marking machine, Shenyang SEPBASE technology Co., Ltd., China). The laser power and frequency were set to 400 mW and 20 kHz, respectively, and the laser beam size was 10 μm with a scan rate up to 0.8 m s^{-1}. The configuration parameters of the patterned MXene electrodes are: length: 14.0 mm, width: ~660 μm, thickness: ~200 nm, interspace: ~840 μm. Finally, m-$HSbP_2O_8$ dispersion was dropped onto the spaces between the patterned electrodes and dried at room temperature to obtain m-$HSbP_2O_8$-MXene MSCs.

The fabrication of Nafion- and GO-MXene MSCs was similar to that of m-$HSbP_2O_8$-

MXene MSCs, where Nafion and GO dispersions were used, respectively, instead of m-$HSbP_2O_8$ dispersion. The H_2SO_4- and H_2SO_4 /PVA-MXene MSCs were fabricated by casting H_2SO_4 (3 M) and H_2SO_4 (3 M)/PVA electrolytes onto the patterned MXene electrodes, respectively. The H_2SO_4 (3 M)/PVA gel electrolyte was synthesized by slowly adding concentrated H_2SO_4 (14.3 g) to a PVA gel (13.5 wt%) at room temperature. The PVA gel was obtained by dissolving PVA (5.5 g) in H_2O (35.2 g) at 90 °C with stirring until the solution was clear.

Electrochemical measurements

The electrochemical behaviors of m-$HSbP_2O_8$, Nafion and GO membranes, H_2SO_4 (3 M), and H_2SO_4 (3 M)/PVA electrolytes and MSCs were measured through the electrochemical workstation (Autolab M204, Metrohm, PGSTAT204) at room temperature. To determine the electrochemical stability of m-$HSbP_2O_8$ membrane and 3.0 M H_2SO_4 solution electrolyte, they were measured in a home-made three-electrode cell using LSV at a scan rate of 2 mV s^{-1}, in which Ti plates (purity: 99.99%) were used as the working and counter electrodes, and Pt wire (purity: 99.99%) as the reference electrode. The electrochemical stability window was selected when the current density was lower than 10^{-2} mA cm^{-2}. The cyclic voltammetry (CV) tests were carried out at 30 – 500 mV s^{-1} and the galvanostatic charge-discharge (GCD) curves were obtained at the current densities of 76 to 304 μA cm^{-2} to characterize the performances of MSCs. In order to quickly achieve water adsorption-desorption equilibrium for m-$HSbP_2O_8$, Nafion and GO membranes as electrolytes, water droplets were added to the surface of the MSCs before electrochemical measurements. The ionic conductivities of H_2SO_4 (3

M) and H_2SO_4 (3 M)/PVA were measured by the conductivity meter (S230 Sevencompact™, Mettler Toledo).

Calculations of the performances of MSCs

The specific areal capacitance (C_{CV} and C_{GCD}) of MSCs was calculated from the CV and GCD curves according to the equations:

$$C_{\mathrm{CV}} = \frac{1}{v \times \Delta V \times A} \int I(V)\, dV \quad (1)$$

$$C_{\mathrm{GCD}} = \frac{I \times \Delta t}{A \Delta V} \quad (2)$$

where A (cm^{-2}), v (V s^{-1}), ΔV (V), Δt (s) and I (A) represent geometry area, scan rate, voltage window, discharge time and current, respectively.

The volumetric capacitance (C_V) of MSCs was calculated from the GCD curves based on the thickness (H, cm) of MXene electrodes according to the equation:

$$C_{\mathrm{V}} = \frac{C_{\mathrm{GCD}}}{H} \quad (3)$$

The volumetric energy density (E_V) and power density (P_V) of MSCs were calculated from the GCD curves according to the equations:

$$E_V = \frac{1}{2 \times 3600} C_{\mathrm{V}} \times \Delta V^2 \quad (4)$$

$$P_{\mathrm{V}} = \frac{3600 E_{\mathrm{V}}}{\Delta t} \quad (5)$$

Characterization of m-$HSbP_2O_8$-MXene MSCs

The thicknesses of the MXene electrodes in the MSCs were measured using a surface profiler (KLA-Tencor P7). To reveal the cross-sectional structure of m-$HSbP_2O_8$ membrane electrolyte and MXene electrodes, thick m-$HSbP_2O_8$ membrane and MXene membrane were fabricated separately with the same method as the fabrication of m-$HSbP_2O_8$-MXene MSCs and characterized with SEM (Verios G4 UC).

Supplementary Figures

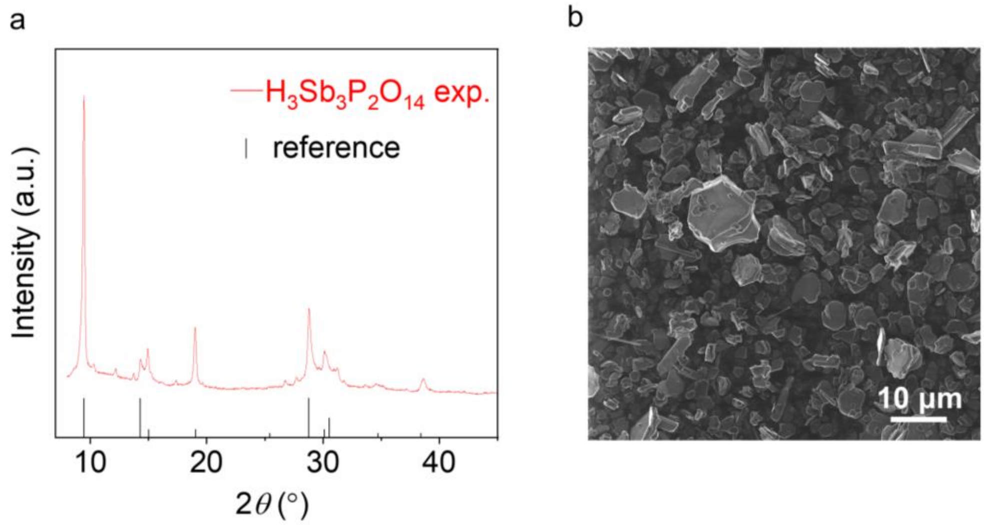

Supplementary Figure 1. a, The XRD pattern of bulk $H_3Sb_3P_2O_{14}$. The reference XRD pattern of bulk $H_3Sb_3P_2O_{14}$ was also presented. **b,** HIM image of bulk $H_3Sb_3P_2O_{14}$ crystals.

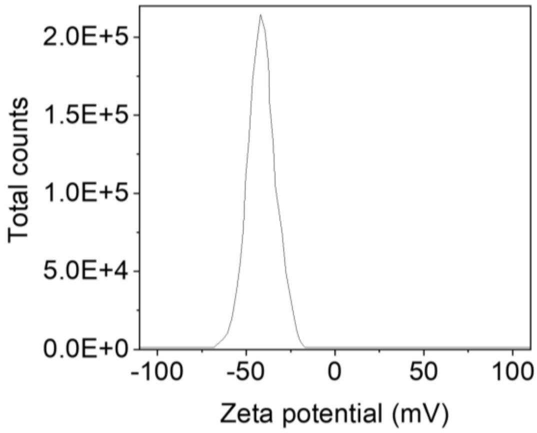

Supplementary Figure 2. Zeta potential of m-$H_3Sb_3P_2O_{14}$ nanosheet aqueous dispersion, showing a value of -41.0 mV at pH = 2.67.

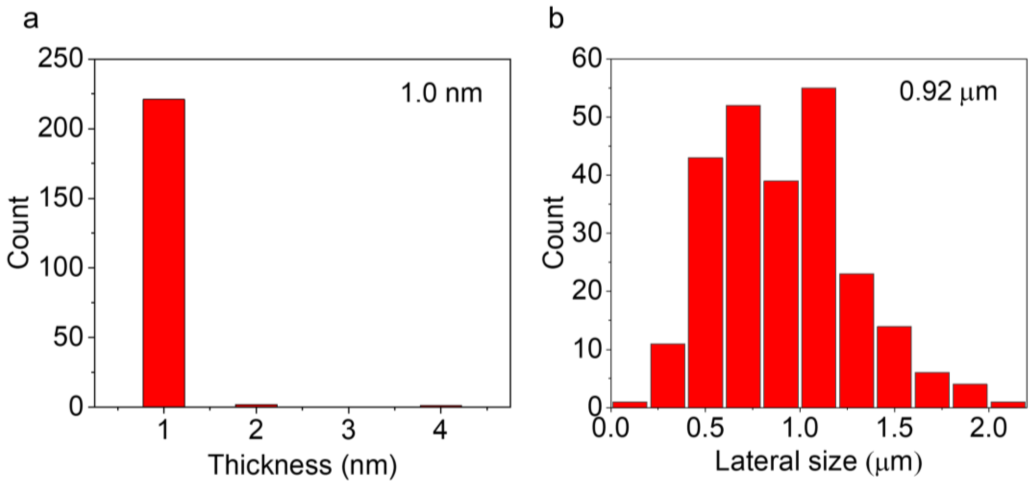

Supplementary Figure 3. The distributions of thickness (**a**) and lateral size (**b**) of $H_3Sb_3P_2O_{14}$ nanosheets obtained at a relative centrifugal force of 13000 g. The average thickness and lateral size are shown in the upper corner of each figure.

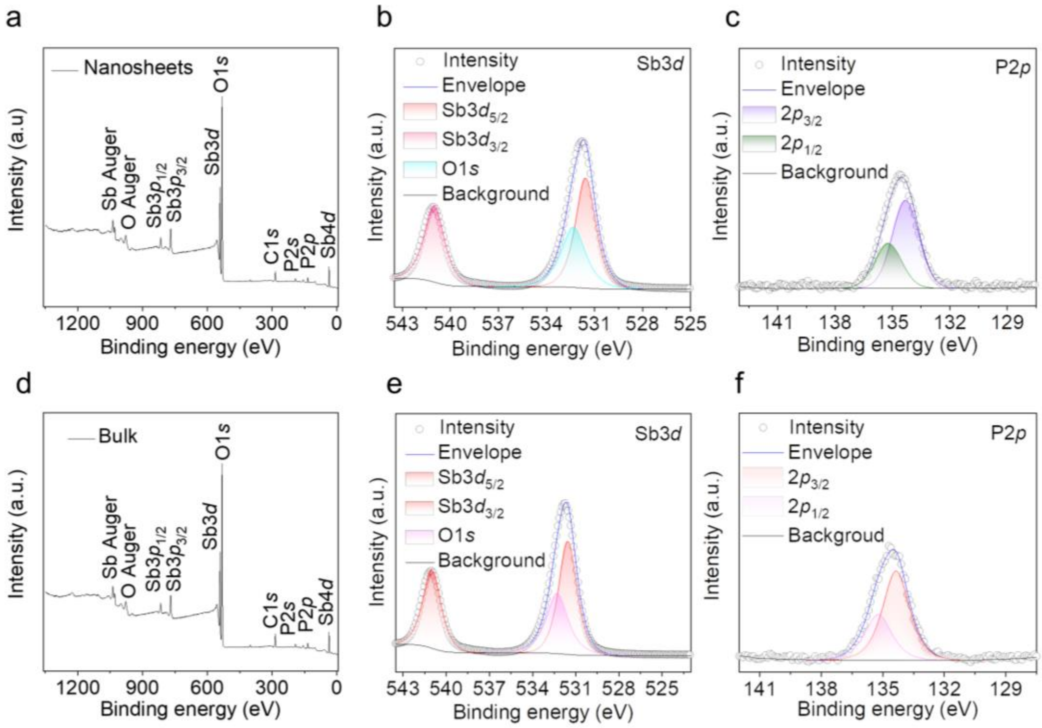

Supplementary Figure 4. XPS spectra of m-$H_3Sb_3P_2O_{14}$ nanosheets and bulk $H_3Sb_3P_2O_{14}$. a-c, Survey XPS spectrum (**a**) and Sb 3*d* (**b**) and P 2*p* (**c**) XPS spectra of m-$H_3Sb_3P_2O_{14}$ nanosheets. The Sb 3*d* spectrum overlaps the spectrum of O 1*s*. The deconvolved peaks at 531.6 eV and 532.3 eV correspond to Sb $3d_{5/2}$ and O 1*s* peaks,

respectively. The P $2p_{3/2}$ and P $2p_{1/2}$ peaks are located at 134.3 eV and 135.1 eV, respectively. **d-f,** Survey XPS spectrum (**d**) and Sb 3*d* (**e**) and P 2*p* (**f**) XPS spectra of $H_3Sb_3P_2O_{14}$ bulk. The deconvolved peaks at 531.5 eV and 532.3 eV correspond to Sb $3d_{5/2}$ and O 1*s* peaks, respectively. The P $2p_{3/2}$ and P $2p_{1/2}$ are located at 134.3 eV and 135.2 eV, respectively. According to the positions of Sb, P and O peaks of $H_3Sb_3P_2O_{14}$ nanosheets and bulk, the chemical valent states of Sb, P and O elements in nanosheets are almost the same as those in the bulk.

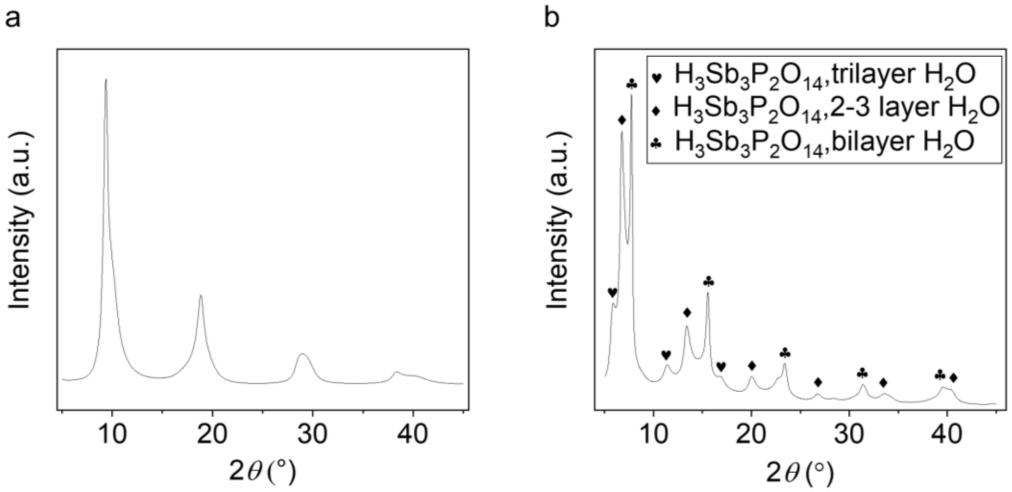

Supplementary Figure 5. The XRD patterns of m-$H_3Sb_3P_2O_{14}$ membrane at 0% RH (**a**) and 100% RH (**b**). At 100% RH, the three XRD peaks in the range of 5° – 10° correspond to three different interlayer distances with a maximum of 1.51 nm, indicating that the nanochannels are inserted up to trilayer water molecules.

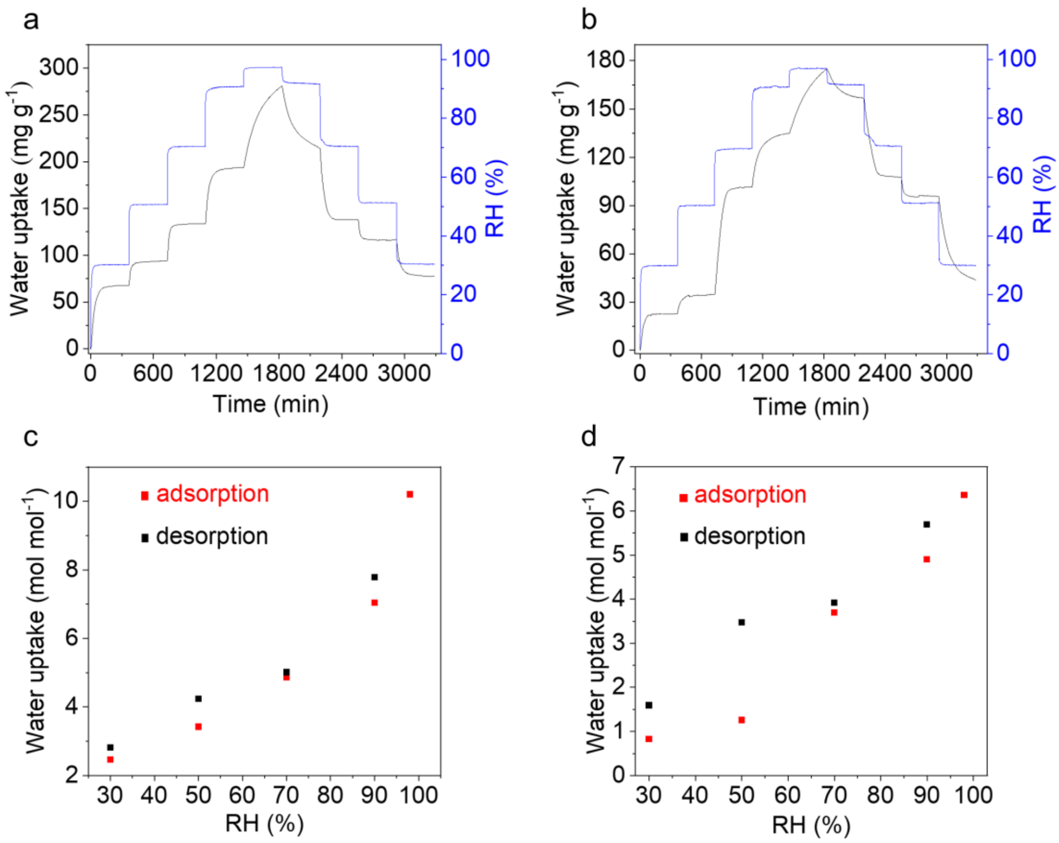

Supplementary Figure 6. a,b, Adsorption-desorption isotherm curves of water vapor in m-$H_3Sb_3P_2O_{14}$ membranes (**a**) and $H_3Sb_3P_2O_{14}$ bulk (**b**) at different RHs and 30 °C. **c,d,** Equilibrium water uptake at different RHs in the process of water vapor adsorption and desorption for m-$H_3Sb_3P_2O_{14}$ membranes (**c**) and $H_3Sb_3P_2O_{14}$ bulk (**d**).

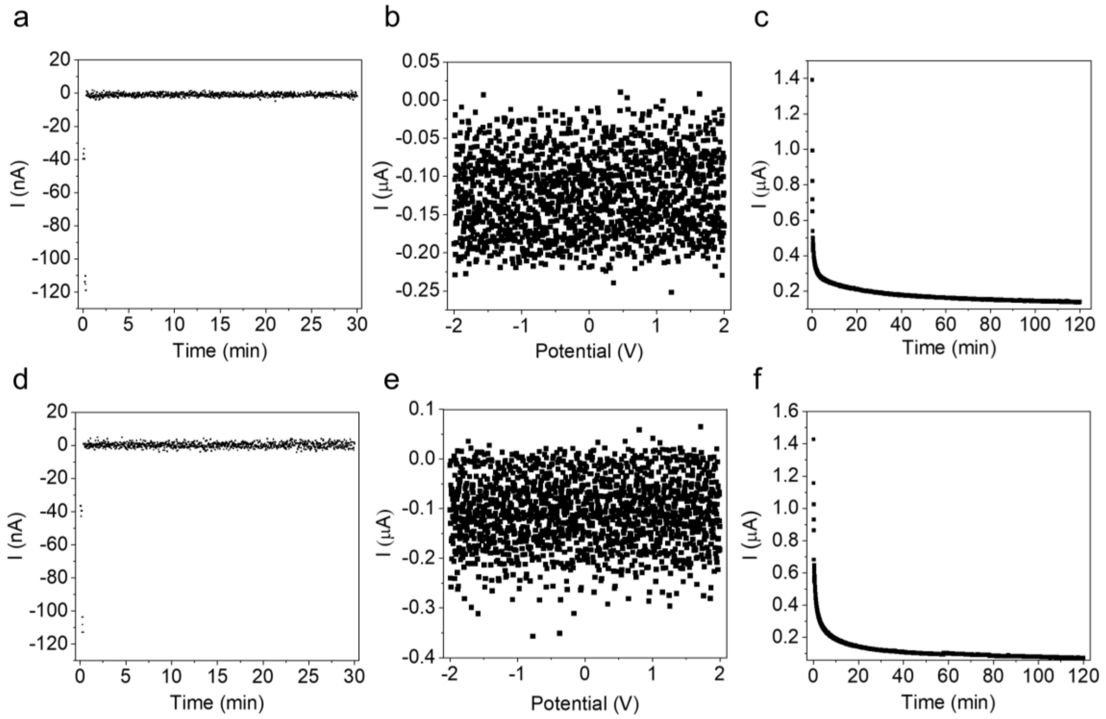

Supplementary Figure 7. Electronic behaviors of m-$H_3Sb_3P_2O_{14}$ membranes and Nafion 117 films. a, Chrono amperometry curve of m-$H_3Sb_3P_2O_{14}$ membranes at a constant bias voltage of 1 V under 0% RH and 90 °C. **b,** LSV curve of m-$H_3Sb_3P_2O_{14}$ membranes at a scanning rate of 0.5 V s^{-1} under 0% RH and 90 °C. **c,** Chrono amperometry curve of m-$H_3Sb_3P_2O_{14}$ membranes at a constant bias voltage of 0.5 V under 100% RH and 60 °C. **d,** Chrono amperometry curve of Nafion 117 films at a constant bias of 1 V under 0% RH and 70 °C. **e,** LSV curve of Nafion 117 films at a scanning rate of 0.5 V s^{-1} under 0% RH and 70 °C. **f,** Chrono amperometry curve of Nafion films at a constant bias voltage of 0.5 V under 100% RH and 60 °C.

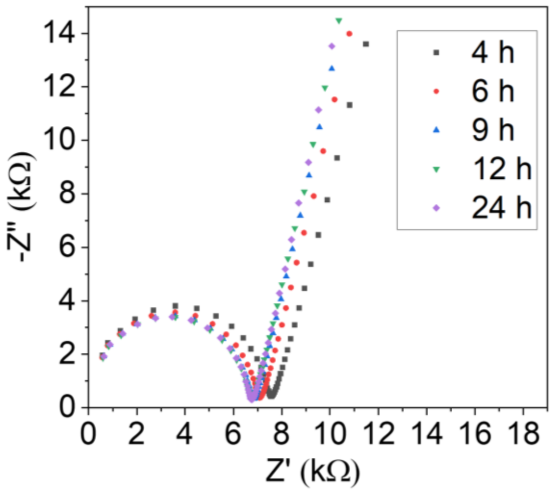

Supplementary Figure 8. Nyquist plots of m-$H_3Sb_3P_2O_{14}$ membranes after storing for different time at 30 °C and RH = 100%, showing that equilibrium was achieved after 12 hours.

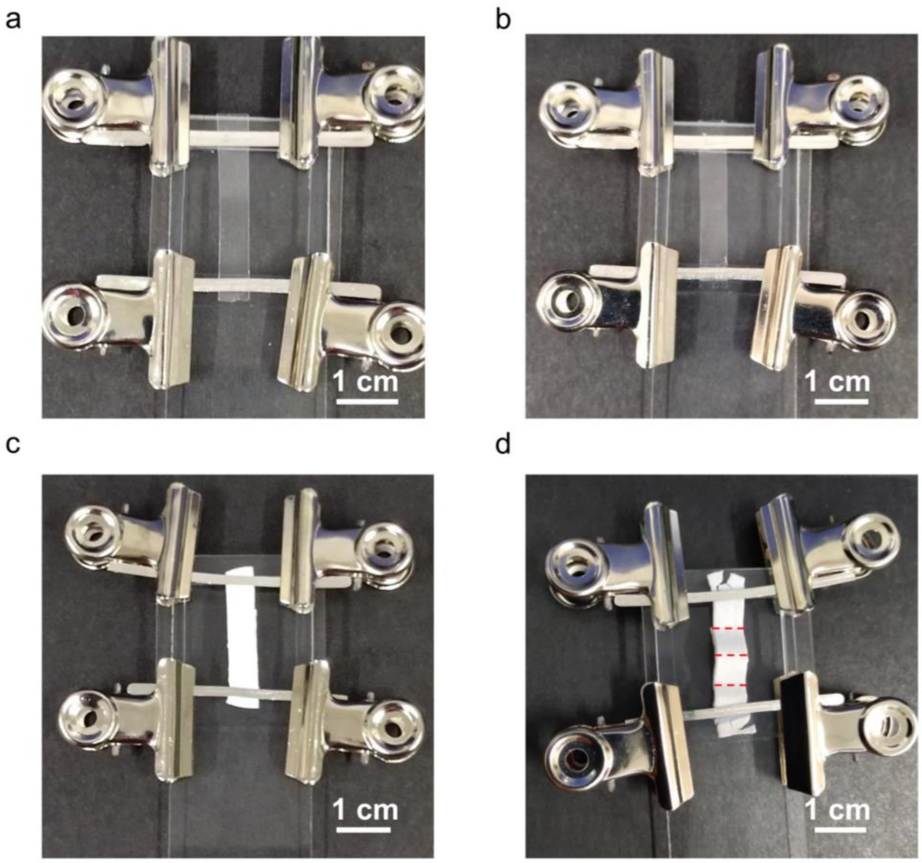

Supplementary Figure 9. a,b, Photographs of m-$H_3Sb_3P_2O_{14}$ membranes before (**a**) and after (**b**) test at 100% RH and 90 °C. **c,d,** Photographs of bulk $H_3Sb_3P_2O_{14}$ pellets

before (**c**) and after (**d**) test at 100% RH and 90 °C. The red dash lines indicate the cracks.

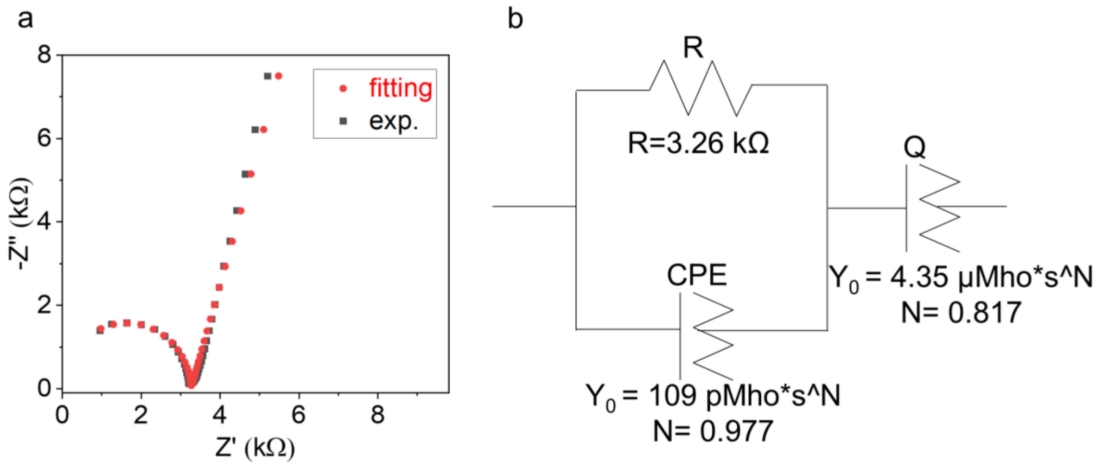

Supplementary Figure 10. a, Nyquist plot of m-$H_3Sb_3P_2O_{14}$ membranes at 90 °C and 100% RH. A semicircle at high frequencies and an incline spur at low frequencies represent the proton transfer impedance and the pile-up of protons at the platinum electrode surface, respectively, which are typical impedance behaviors of proton conductors. **b,** The equivalent circuit for fitting the membranes impedance behaviors. The R, constant phase element (CPE) and Q elements correspond to the proton transfer resistance, capacitive contribution between the nanosheets in m-$H_3Sb_3P_2O_{14}$ membranes and m-$H_3Sb_3P_2O_{14}$ membrane/platinum electrode interface dielectric capacitance, respectively.

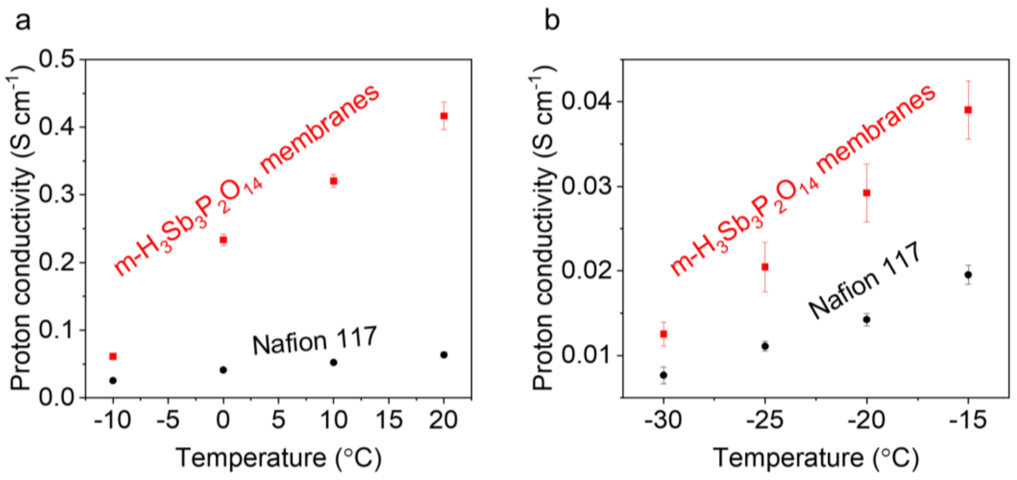

Supplementary Figure 11. The proton conductivities of m-$H_3Sb_3P_2O_{14}$ membranes and Nafion117 membranes at temperatures of 20 to -10 °C (**a**) and -15 to -30 °C (**b**). Error bars represent standard deviations.

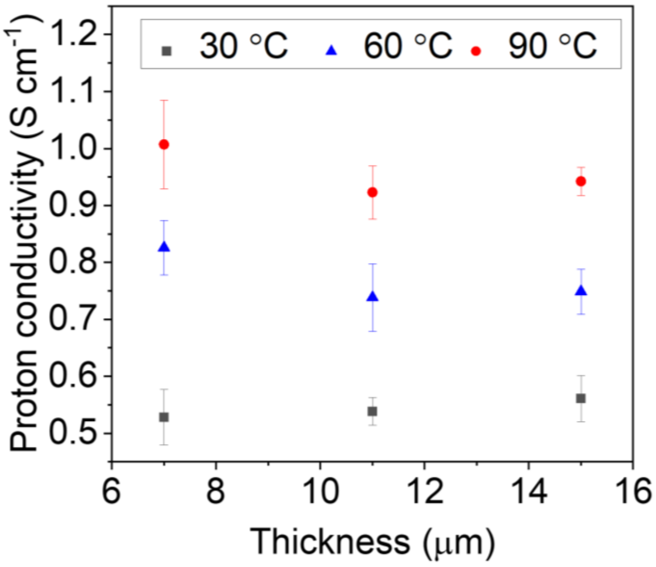

Supplementary Figure 12. The relationships between the proton conductivities and the thicknesses of m-$H_3Sb_3P_2O_{14}$ membranes at 100% RH and different temperatures. Error bars represent standard deviations.

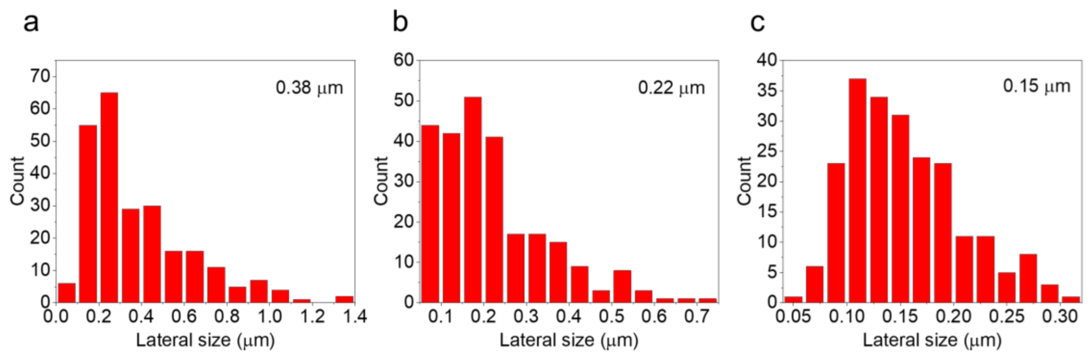

Supplementary Figure 13. The lateral size distributions of m-$H_3Sb_3P_2O_{14}$ nanosheets synthesized at ultrasonic power and time of 160 W and 5 mins (**a**), 160 W and 20 mins (**b**), and 320 W and 20 mins (**c**). The average lateral sizes are shown in the upper corner of each figure.

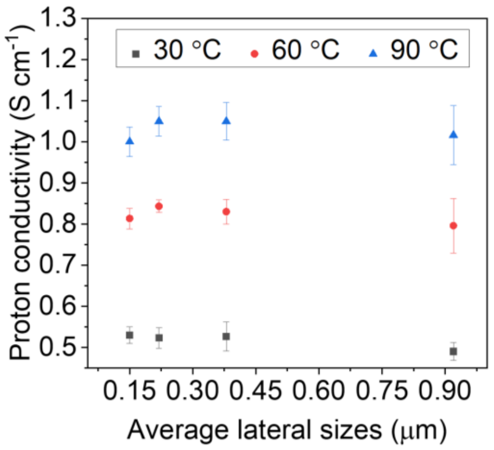

Supplementary Figure 14. The relationships between the proton conductivities of m-$H_3Sb_3P_2O_{14}$ membranes and the lateral sizes of m-$H_3Sb_3P_2O_{14}$ nanosheets at 100% RH and different temperatures. Error bars represent standard deviations.

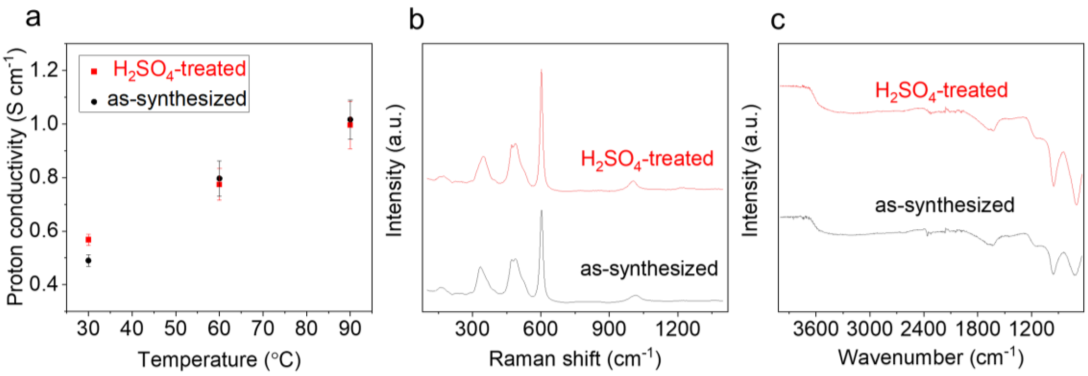

Supplementary Figure 15. The proton conductivities (**a**), Raman spectra (**b**), and FT-IR spectra (**c**) of the m-$H_3Sb_3P_2O_{14}$ membranes before and after immersing in 10 M H_2SO_4 for 12 days. Error bars represent standard deviations.

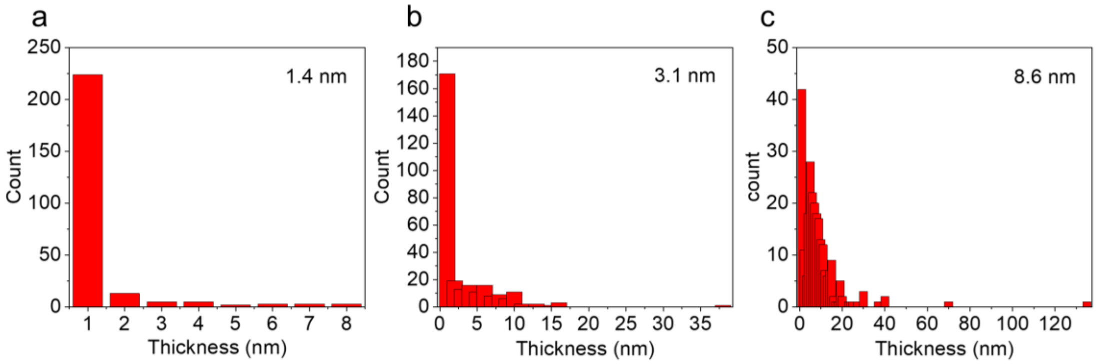

Supplementary Figure 16. The thickness distributions of the $H_3Sb_3P_2O_{14}$ nanosheets obtained at a relative centrifugal force of 7312 – 13000 g (**a**), 3250 – 7312 g (**b**), and 1106 – 3250 g (**c**). The average thicknesses are shown in the upper corner of each figure.

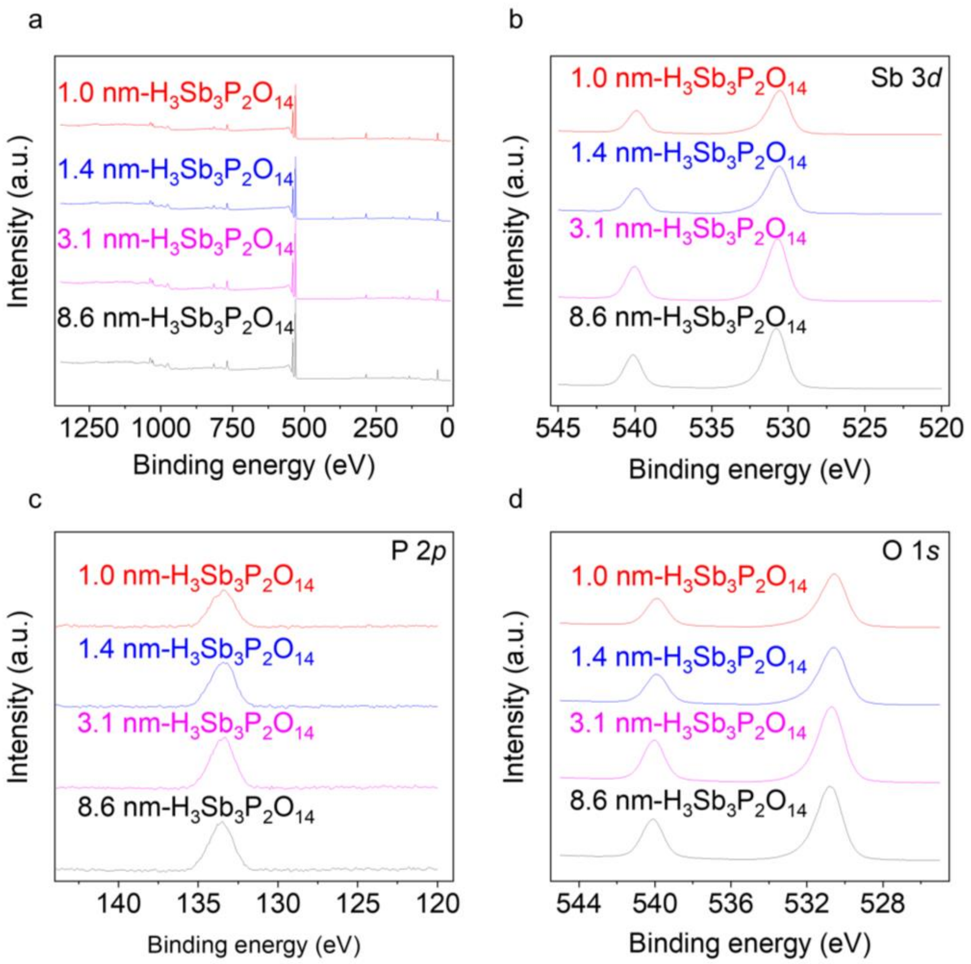

Supplementary Figure 17. Survey XPS spectra (**a**), and Sb 3*d* (**b**), P 2*p* (**c**) and O 1*s* (**d**) XPS spectra of 1.0 nm-, 1.4 nm-, 3.1 nm-, and 8.6 nm-$H_3Sb_3P_2O_{14}$ membranes.

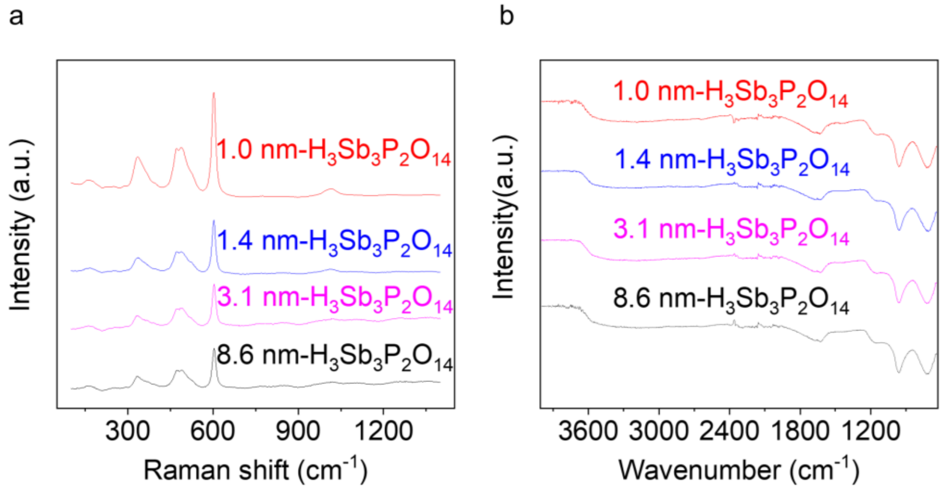

Supplementary Figure 18. Raman (**a**) and FT-IR (**b**) spectra of 1.0 nm-, 1.4 nm-, 3.1 nm-, and 8.6 nm-$H_3Sb_3P_2O_{14}$ membranes.

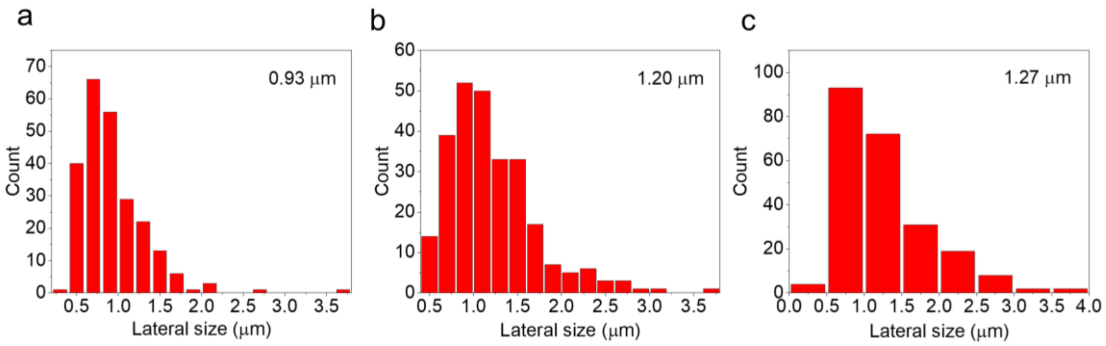

Supplementary Figure 19. The lateral size distributions of $H_3Sb_3P_2O_{14}$ nanosheets obtained at a relative centrifugal force of 7312 – 13000 g (**a**), 3250 – 7312 g (**b**), and 1106 – 3250 g (**c**). The average lateral sizes are shown in the upper corner of each figure.

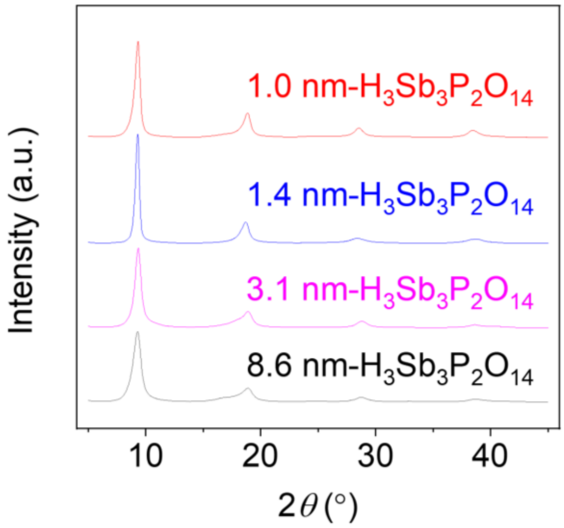

Supplementary Figure 20. XRD patterns of 1.0 nm-, 1.4 nm-, 3.1 nm-, and 8.6 nm-$H_3Sb_3P_2O_{14}$ membranes, showing highly oriented structure along the (00*l*) crystal plane.

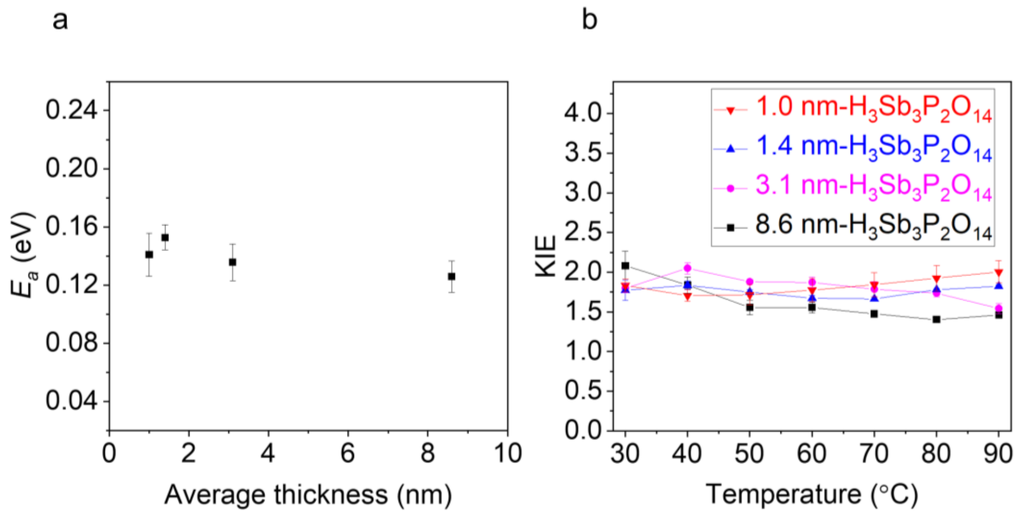

Supplementary Figure 21. Activation energies (**a**) and KIEs (**b**) of the membranes assembled by $H_3Sb_3P_2O_{14}$ nanosheets with different average thicknesses at 100% RH. Error bars represent standard deviations.

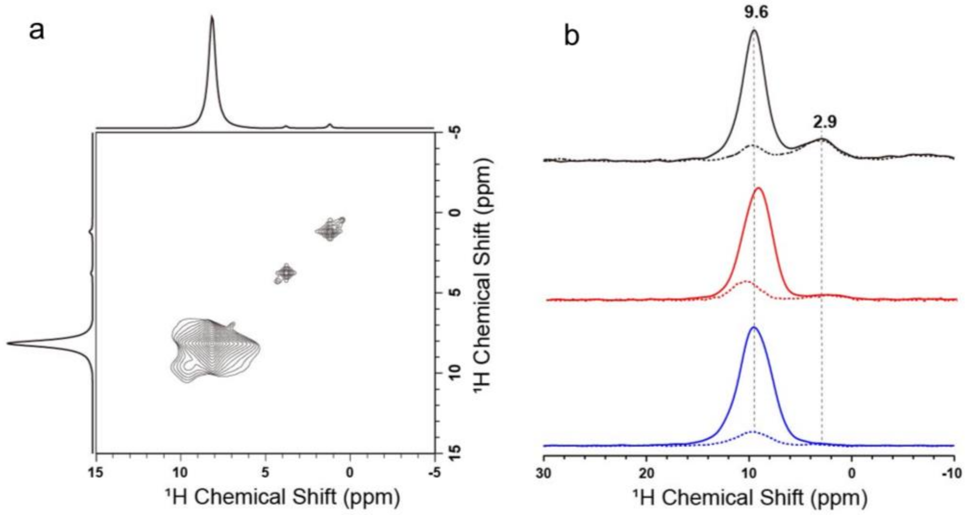

Supplementary Figure 22. a, 2D ^1H-^1H EXSY NMR spectrum of bulk $H_3Sb_3P_2O_{14}$, with mixing time of 10.0 ms. **b,** ^1H PFG NMR spectra of bulk $H_3Sb_3P_2O_{14}$ (black line), m-$H_3Sb_3P_2O_{14}$ membrane (red line) and m-$H_3Sb_3P_2O_{14}$ nanosheets (bule line). The solid and dashed lines indicate diffusion time of 0 and 8.0 ms, respectively. All the samples were kept at 100% RH.

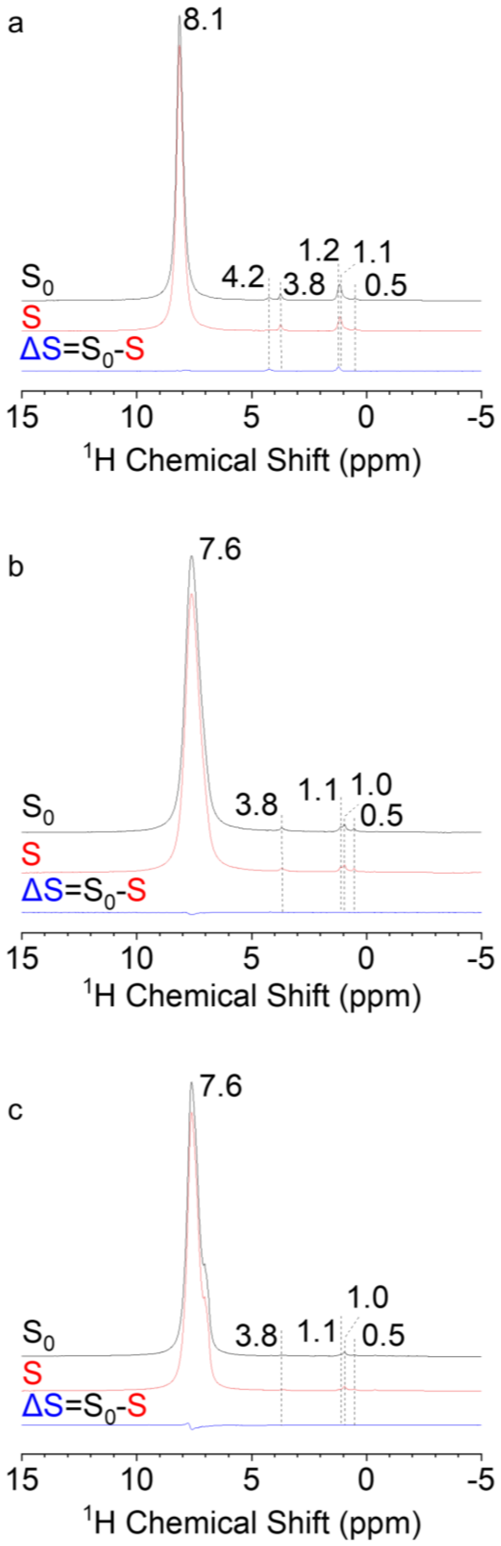

Supplementary Figure 23. $^{1}H\{^{31}P\}$ S-REDOR NMR spectra of $H_3Sb_3P_2O_{14}$ bulk (**a**), m-$H_3Sb_3P_2O_{14}$ membrane (**b**), and m-$H_3Sb_3P_2O_{14}$ nanosheets (**c**). The black lines are reference spectra (S_0) in S-REDOR experiments. The red lines are the ^{1}H spectra with

^{1}H-^{31}P dipolar dephasing (denoted as S). The blue lines are the difference spectra (ΔS) obtained by subtracting S from S_0, i.e., $S_0 - S$. The recoupling times in (**a**), (**b**) and (**c**) are 5.0 ms.

Supplementary Discussion 1

As shown in Supplementary Figure 22a, the 2D ^{1}H-^{1}H EXSY NMR spectrum of the $H_3Sb_3P_2O_{14}$ bulk sample generated no cross peak for signals at 8.1, 4.2, 3.8 and 1.2 ppm. This result indicates that the ^{1}H species with chemical shift < 5 ppm do not undergo chemical exchange with the dominating ^{1}H signal at 8.1 ppm, and these ^{1}H species are spatially far away from each other.

Due to the lack of MAS, the ^{1}H peaks in PFG NMR experiments are broaden, and thus the peak positions are not strictly consistent with the chemical shifts (Supplementary Fig. 22b). But for all three samples, two ^{1}H peaks at ~9.6 and ~2.9 ppm were resolved, corresponding to the ^{1}H MAS NMR signals at ~8 ppm and < 5 ppm, respectively. As clearly shown in the ^{1}H PFG spectra, intensities of the ^{1}H peaks at ~9.6 ppm decreased significantly as the diffusion time increased from 0 to 8.0 ms, indicating a relatively fast self-diffusion of the corresponding ^{1}H species. On the other hand, the ^{1}H peaks at ~2.9 ppm remained unchanged with increasing diffusion time, indicating that the self-diffusion of the corresponding ^{1}H species was not observed, i.e., these were inactive protons difficult to move or be exchanged. It is apparent that the unexchangeable inactive protons in $H_3Sb_3P_2O_{14}$ bulk sample had a much higher relative intensity. In contrast, the same ^{1}H signals at 2.9 ppm were noticeably weaker in the membrane sample and it almost disappeared in the nanosheet sample. The difference in

relative intensity of 2.9 ppm ^{1}H signals suggested that there was a noticeable amount of protons trapped in the $Sb_3P_2O_{14}^{3-}$ framework of $H_3Sb_3P_2O_{14}$ bulk sample, but most protons were released from the framework in the membrane and nanosheet samples. Further evidence showing the connection between protons and the $Sb_3P_2O_{14}^{3-}$ framework in the bulk samples was presented in Supplementary Figure 23.

In $^{1}H\{^{31}P\}$ S-REDOR NMR experiments, the intensity of ^{1}H signal is decreased if the corresponding protons are fixed in the crystalline and, at the same time, in proximity with ^{31}P atom(s). In this condition, the ^{1}H-^{31}P dipolar coupling can be recoupled in S-REDOR experiments, resulting in the loss of signal intensity of the fixed protons. For protons with high mobility, the ^{1}H-^{31}P dipolar coupling is averaged out by the fast proton motion, thus the corresponding signal intensity is unchanged. The decreased signal intensity can be revealed by subtracting the spectra (S) from the reference spectra (S_0), and the result is a difference spectrum denoted as ΔS, i.e., $\Delta S = S_0 - S$.

In $H_3Sb_3P_2O_{14}$ bulk sample (Supplementary Fig. 23a), the ΔS spectrum showed two signals at 4.2 and 1.2 ppm, indicating that the corresponding protons were fixed to the $Sb_3P_2O_{14}^{3-}$ framework. On the contrary, the ^{1}H signal at 8.1 ppm was unchanged, only slightly distorted baseline was observed, originated from the slight phase difference of the strong signals (which were truncated in Supplementary Figure 23). On the other hand, in the S_0 spectra of both m-$H_3Sb_3P_2O_{14}$ membrane and nanosheet samples (Supplementary Fig. 23b,c), the signals at 4.2 and 1.2 ppm were not observed and no peak was presented in the ΔS spectra. It can be concluded that the ^{1}H species at 4.2 and 1.2 ppm were less mobile, and the ^{1}H species at 8.1 or 7.6 ppm were in fast motion (fast

exchange, hopping, transportation, etc.). Moreover, compared to the $H_3Sb_3P_2O_{14}$ bulk sample, the protons in m-$H_3Sb_3P_2O_{14}$ membrane and nanosheet samples possess higher mobility as shown in ΔS spectra.

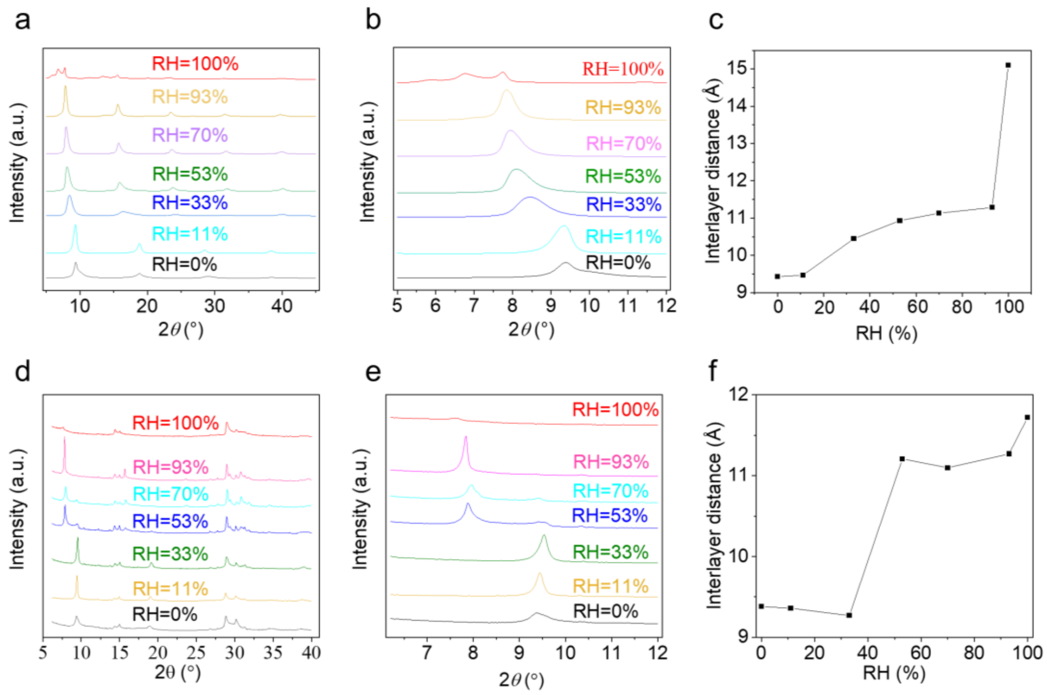

Supplementary Figure 24. a,d, The XRD patterns of m-$H_3Sb_3P_2O_{14}$ membranes (**a**) and bulk $H_3Sb_3P_2O_{14}$ pellets (**d**) at different RHs. **b,e,** Zoom-in XRD patterns in the range of 5° to 12° for m-$H_3Sb_3P_2O_{14}$ membranes (**b**) and bulk $H_3Sb_3P_2O_{14}$ pellets (**e**). **c,f,** RH-dependent interlayer distances of m-$H_3Sb_3P_2O_{14}$ membranes (**c**) and bulk $H_3Sb_3P_2O_{14}$ pellets (**f**) at different RHs extracted from the XRD data. For 100% RH, the largest interlayer distance was given.

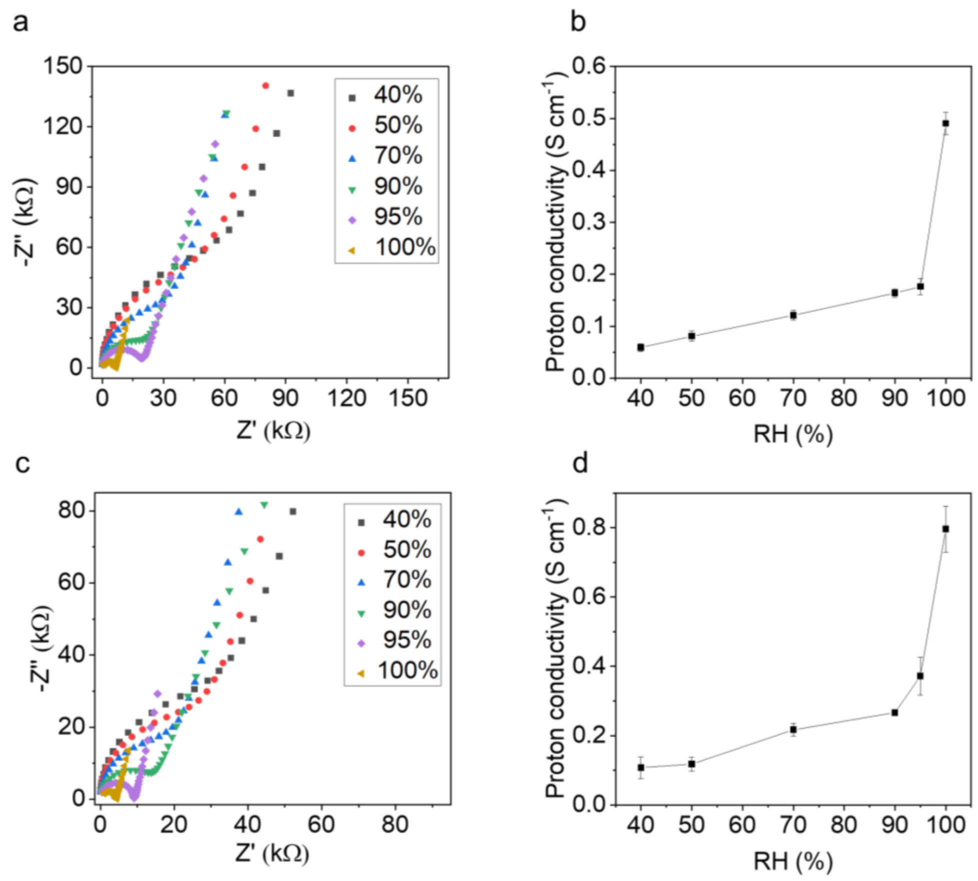

Supplementary Figure 25. a, Nyquist plots of m-$H_3Sb_3P_2O_{14}$ membranes at different RHs and 30 °C. **b,** RH-dependent proton conductivities of m-$H_3Sb_3P_2O_{14}$ membranes at 30 °C. **c,** Nyquist plots of m-$H_3Sb_3P_2O_{14}$ membranes at different RHs and 60 °C. **d,** RH-dependent proton conductivities of m-$H_3Sb_3P_2O_{14}$ membranes at 60 °C. Error bars represent standard deviations.

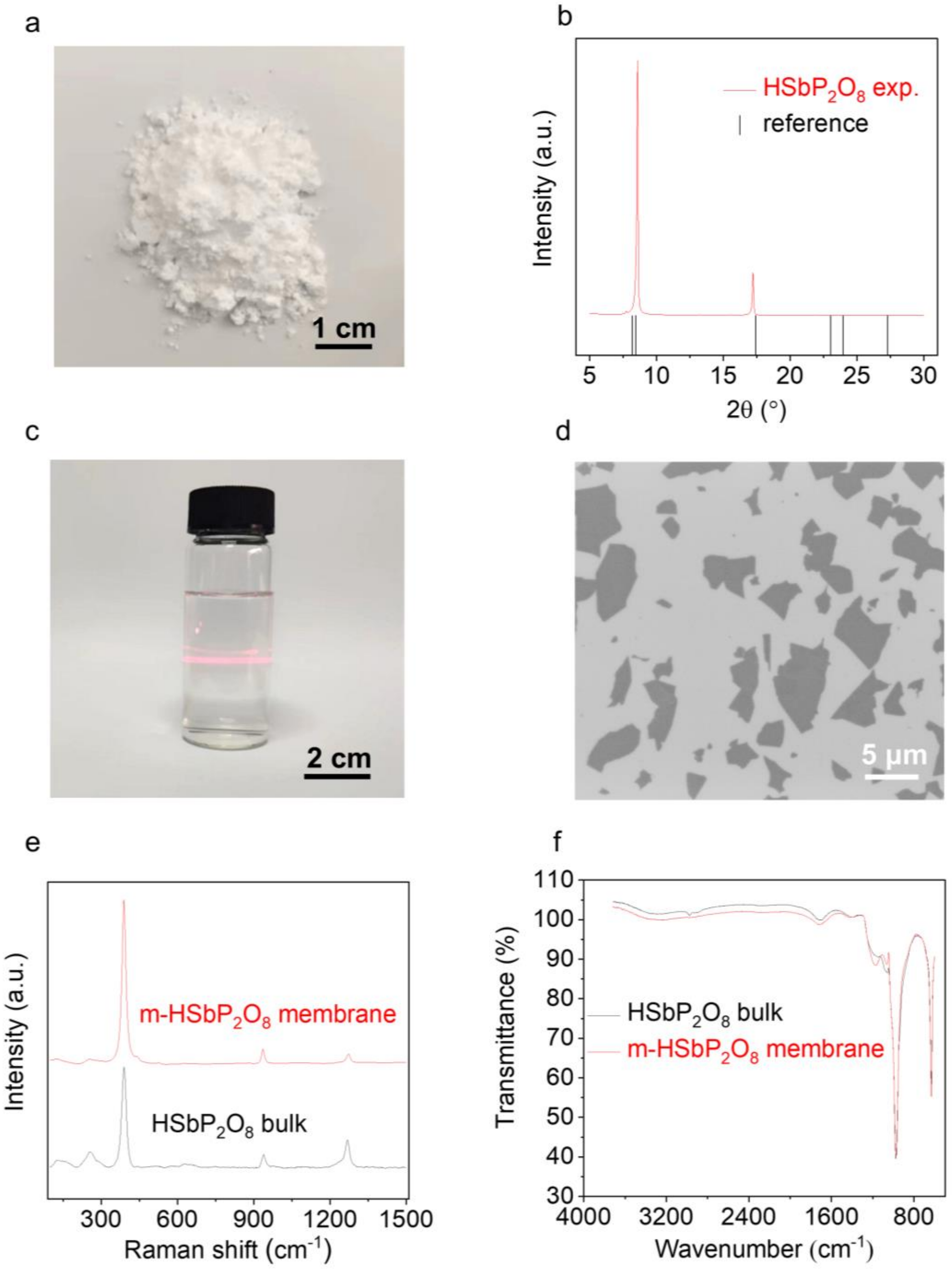

Supplementary Figure 26. a, Photograph of bulk $HSbP_2O_8$. **b,** XRD pattern of bulk $HSbP_2O_8$. The reference XRD pattern of bulk $HSbP_2O_8$ was also presented. **c,** Photograph of m-$HSbP_2O_8$ nanosheets aqueous dispersion with the Tyndall effect. **d,** SEM image of m-$HSbP_2O_8$ nanosheets on SiO_2/Si substrate. **e,f,** Raman spectra (**e**) and FI-IR spectra (**f**) of m-$HSbP_2O_8$ membrane and bulk $HSbP_2O_8$.

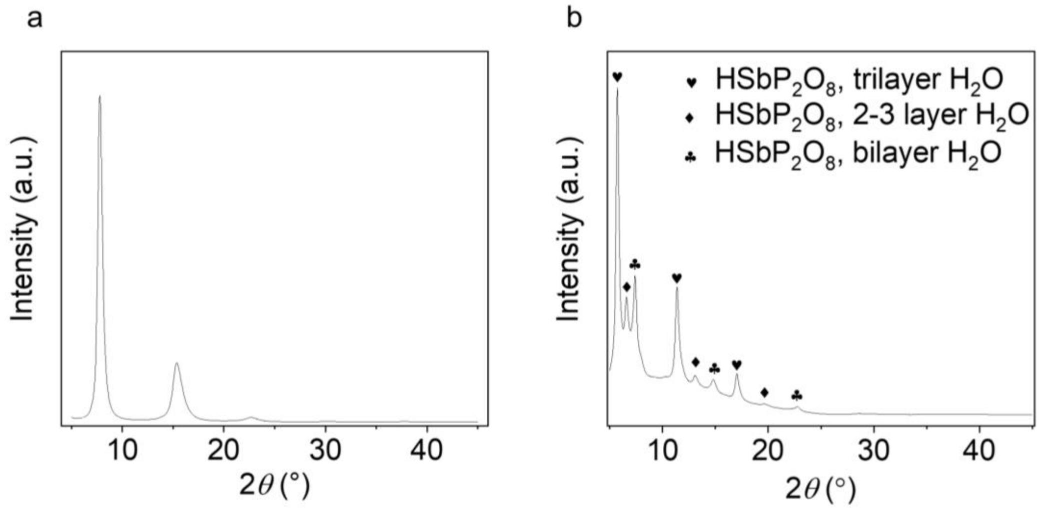

Supplementary Figure 27. The XRD patterns of m-$HSbP_2O_8$ membranes at 0% RH (**a**) and 100% RH (**b**). Similar to m-$H_3Sb_3P_2O_{14}$ membrane, there are three XRD peaks in the range of 5° – 10° at 100% RH, corresponding to three interlayer distances with a maximum of 1.54 nm, which indicates that the nanochannels are inserted up to trilayer water molecules.

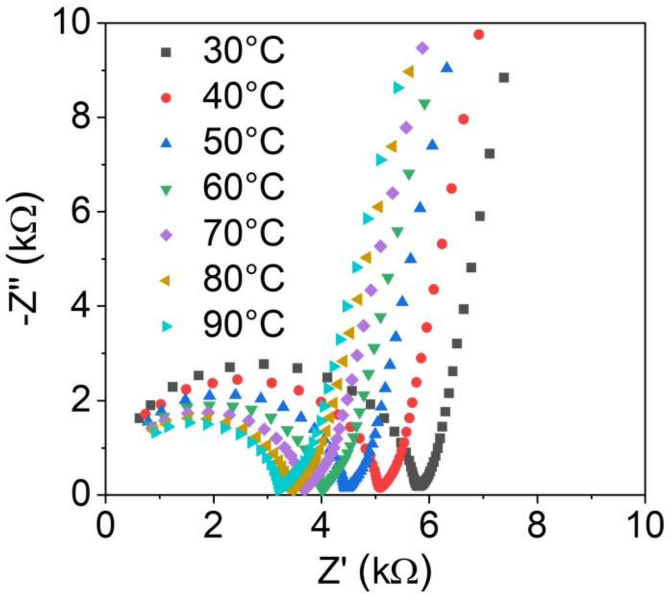

Supplementary Figure 28. Nyquist plots of m-$HSbP_2O_8$ membranes at different temperatures and 100% RH.

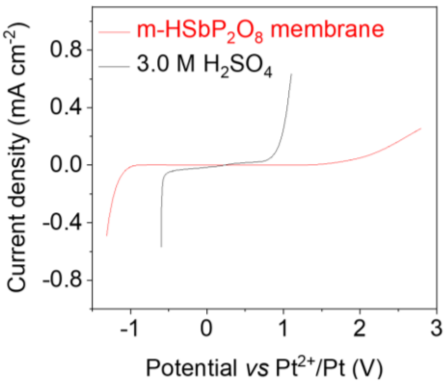

Supplementary Figure 29. The LSV curves of fully hydrated m-$HSbP_2O_8$ membrane and 3.0 M H_2SO_4 solution at the polarization scanning of 2 mV s^{-1}.

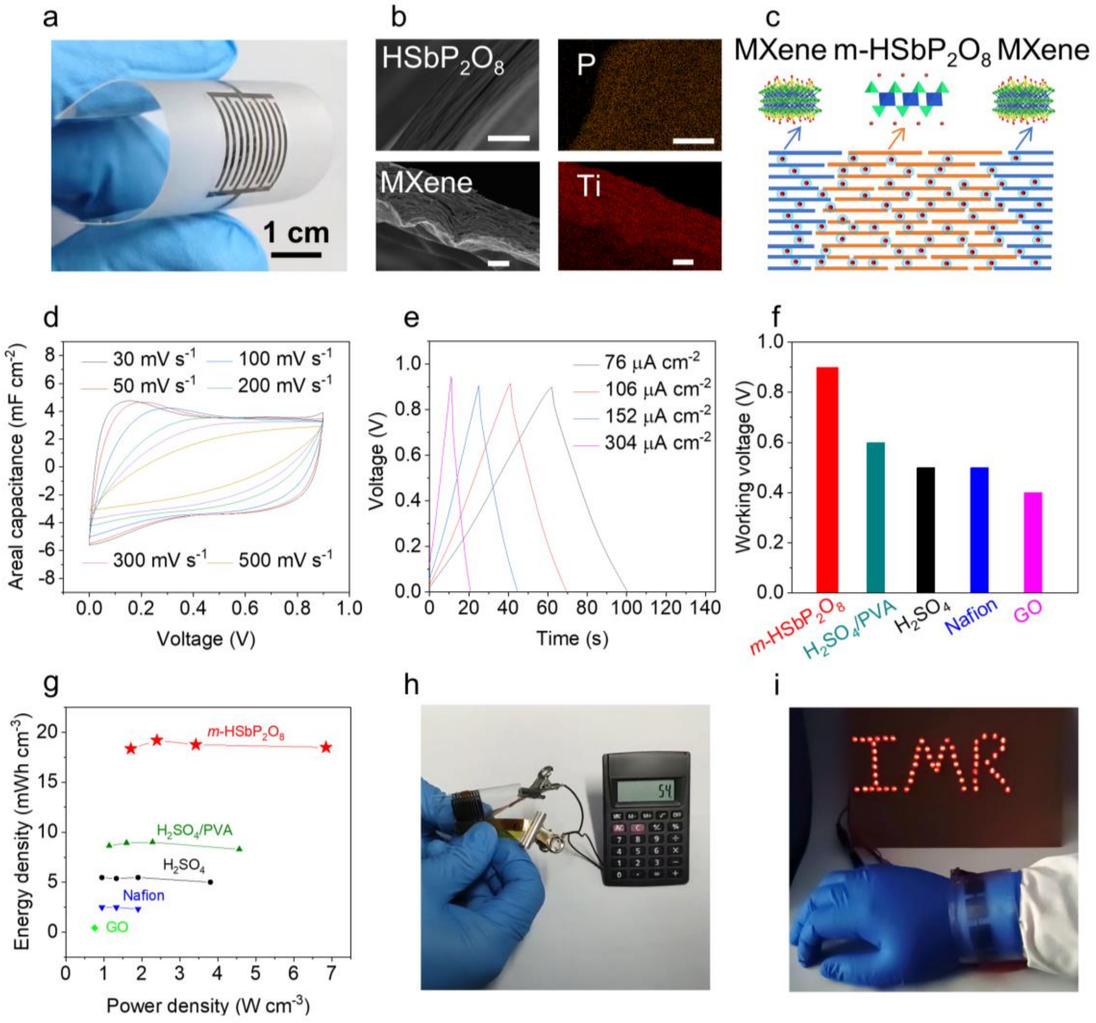

Supplementary Figure 30. Demonstration of all-2D flexible solid-state m-$HSbP_2O_8$-MXene MSCs. a, Photograph of a m-$HSbP_2O_8$-MXene MSC, showing good

flexibility. **b**, Cross-sectional SEM images and the corresponding EDS mappings of the m-$HSbP_2O_8$ membrane and MXene membrane. Scale bar, 5 μm. **c**, Illustration of the interconnected nanochannels in m-$HSbP_2O_8$-MXene MSCs. The red balls surrounded by blue rings represent hydrated protons. **d**, Typical CV curves of m-$HSbP_2O_8$-MXene MSCs at the scan rate from 30 mV s^{-1} to 500 mV s^{-1}. **e**, The GCD curves of the m-$HSbP_2O_8$-MXene MSCs at different current densities. **f**, Comparison of the operating voltages of m-$HSbP_2O_8$-, H_2SO_4-, H_2SO_4/PVA-, Nafion-, and GO-MXene MSCs. **g**, The Ragone plots showing the volumetric energy and power densities of m-$HSbP_2O_8$-, H_2SO_4-, H_2SO_4/PVA-, Nafion-, and GO-MXene MSCs. **h,i**, Demonstration of the applications of m-$HSbP_2O_8$-MXene MSCs, where five packaging-free devices were connected in series to light up a "IMR" LOGO assembled by 56 LEDs. The "IMR" LOGO in (**i**) is used with permission from the Institute of Metal Research, Chinese Academy of Sciences.

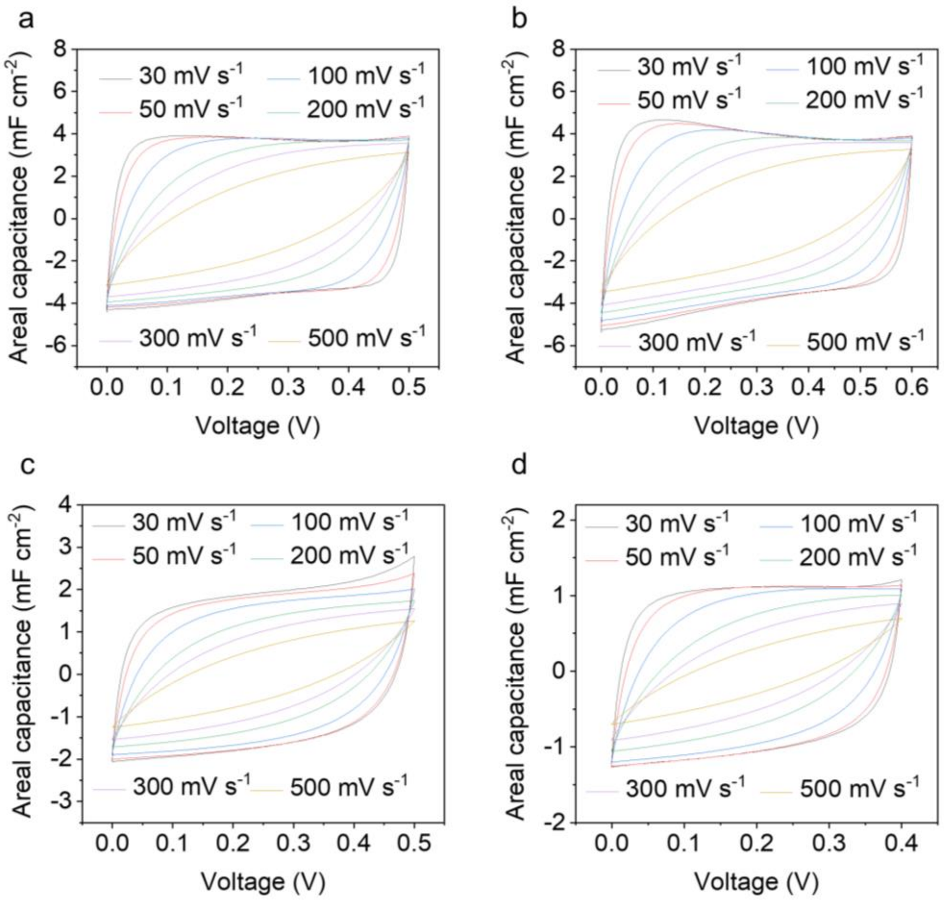

Supplementary Figure 31. The CV curves of H_2SO_4-MXene (**a**), H_2SO_4/PVA-MXene (**b**), Nafion-MXene (**c**), and GO-MXene (**d**) MSCs at the scan rate from 30 to 500 mV s^{-1}.

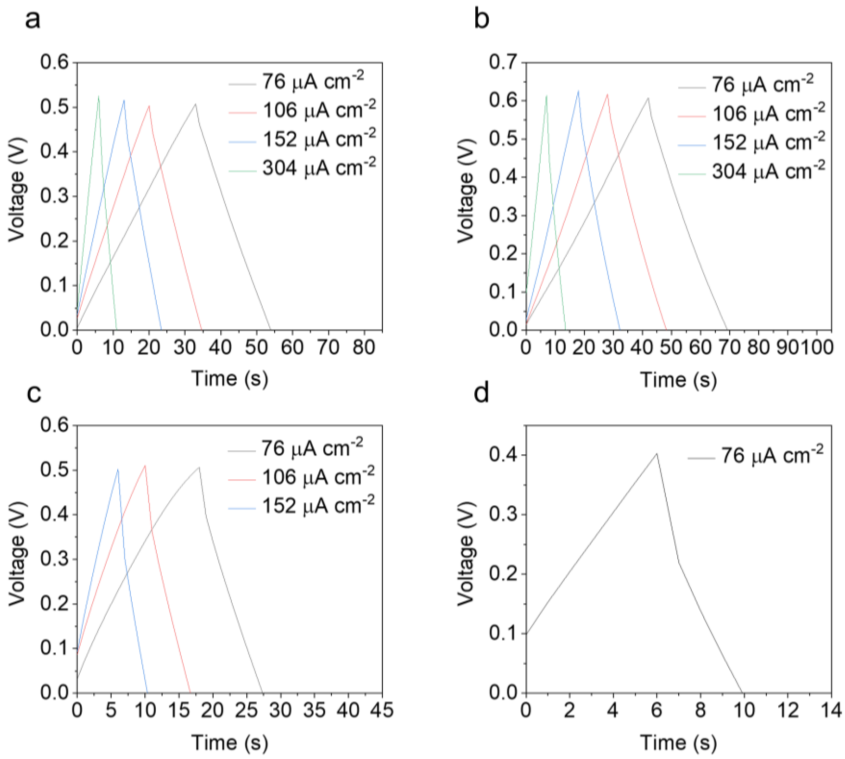

Supplementary Figure 32. The GCD curves of the H_2SO_4-MXene (**a**), H_2SO_4/PVA-MXene (**b**), Nafion-MXene (**c**), and GO-MXene (**d**) MSCs at different current densities.

Supplementary Discussion 2

Taking into account the high proton conductivity and electronic insulating nature of m-$HSbP_2O_8$ membrane, as an example, we demonstrated its practical use as an H^+ solid-state electrolyte and electrode separator in high-safety energy storage devices. The LSV curves show that m-$HSbP_2O_8$ membrane features a significantly broader electrochemical stability window (~2.50 V *vs* Pt^{2+}/Pt) than that of H_2SO_4 aqueous solution (~0.25 V *vs* Pt^{2+}/Pt at 3.0 M) (Supplementary Fig. 29), the most commonly used H^+ electrolyte. We fabricated flexible all-2D H^+ solid-state MSCs by using m-$HSbP_2O_8$ membranes and MXene membranes as solid-state electrolyte (0.49 S cm^{-1} at room temperature) and electrodes, respectively (Supplementary Fig. 30a,b). MXene is

an excellent electrode material with high capacitance and easy to assembled into well-ordered layered membranes[1,3-8]. In such devices, m-$HSbP_2O_8$ and MXene membranes construct interconnected nanochannels with the same orientation (Supplementary Fig. 30b,c), which fully utilize the highly anisotropic structure and proton transport characteristics of both membranes and ensure small obstacles of H^+ transport in the whole devices. For comparison, we also fabricated MSCs with the same electrodes using 3 M H_2SO_4 solution, 3 M H_2SO_4/PVA gel, solid Nafion and GO membranes as electrolytes, respectively, which have ionic conductivities of ~0.71, 0.49, 0.04 and 0.003 S cm^{-1} at room temperature.

Supplementary Figure 30d shows that the m-$HSbP_2O_8$-MXene MSCs exhibit good capacitive behaviors, where the CV curves maintain a rectangular-like shape with a capacitance over 1.9 mF cm^{-2} at a superhigh scan rate of 300 mV s^{-1}, which is similar to the devices using H_2SO_4 and H_2SO_4/PVA as electrolytes (Supplementary Fig. 31a,b). In contrast, the Nafion-MXene and GO-MXene MSCs show poor electrochemical performances (Supplementary Fig. 31c,d). Despite the similar interconnected proton transport nanochannels in GO-MXene MSCs, they show a much lower capacitance of 0.37 mF cm^{-2} at 300 mV s^{-1}. Importantly, the m-$HSbP_2O_8$-MXene MSCs output a high operating voltage (0.9 V) (Supplementary Fig. 30e), which is over 1.5 times larger than those of the MSCs using other electrolytes, ranging from 0.4 V for GO membrane to 0.6 V for H_2SO_4/PVA gels (Supplementary Figs. 30f and 32). As a result, the m-$HSbP_2O_8$-MXene MSCs show similarly high volumetric energy densities of 18.5 – 18.3 mWh cm^{-3} with the corresponding power densities in the range of 1.7 – 6.8 W cm^{-3}

(Supplementary Fig. 30g), which is higher than or comparable to those of the reported typical solid-state MXene-based MSCs[8-10]. These performances are significantly better than those of H_2SO_4-, H_2SO_4/PVA-, Nafion-, and GO-MXene MSCs with the same electrodes. Furthermore, such all-2D MSCs can be used to power the electronic devices even under repeated bending without packaging (Supplementary Fig. 30h,i and Supplementary movie 1), demonstrating the great potential of m-$HSbP_2O_8$ membranes for practical applications in flexible solid-state micro energy storage devices.

Supplementary References

1. Ghidiu, M., Lukatskaya, M. R., Zhao, M.-Q., Gogotsi, Y. & Barsoum, M. W. Conductive two-dimensional titanium carbide 'clay' with high volumetric capacitance. *Nature* **516**, 78–81 (2014).
2. Chen, J., Yao, B. W., Li, C. & Shi, G. Q. An improved Hummers method for eco-friendly synthesis of graphene oxide. *Carbon* **64**, 225–229 (2013).
3. Anasori, B., Lukatskaya, M. R. & Gogotsi, Y. 2D metal carbides and nitrides (MXenes) for energy storage. *Nat. Rev. Mater.* **2**, 16098 (2017).
4. Anasori, B. & Gogotsi, Y. *2D Metal Carbides and Nitrides (MXenes): Structure, Properties and Applications* (Springer International Publishing, 2019).
5. Lukatskaya, M. R. et al. Cation intercalation and high volumetric capacitance of two-dimensional titanium carbide. *Science* **341**, 1502–1505 (2013).
6. VahidMohammadi, A., Rosen, J. & Gogotsi, Y. The world of two-dimensional carbides and nitrides (MXenes). *Science* **372**, eabf1581 (2021).
7. Mu, X. P. et al. Revealing the pseudo-intercalation charge storage mechanism of MXenes in acidic electrolyte. *Adv. Funct. Mater.* **29**, 1902953 (2019).
8. Peng, Y.-Y. et al. All-MXene (2D titanium carbide) solid-state microsupercapacitors for on-chip energy storage. *Energy Environ. Sci.* **9**, 2847–2854 (2016).
9. Wang, S. et al. Monolithic integrated micro-supercapacitors with ultra-high systemic volumetric performance and areal output voltage. *Natl. Sci. Rev.* **10**, nwac271 (2023).

10. Jiang, Q. et al. MXene electrochemical microsupercapacitor integrated with triboelectric nanogenerator as a wearable self-charging power unit. *Nano. Energy* **45**, 266–272 (2018).
