# Supplementary material for: Significant enhancement of proton conductivity in solid acid at the monolayer limit
Source: Nat Commun. 2024 Mar 27;15:2706. doi: 10.1038/s41467-024-46911-7 (PMC10973524; doi:10.1038/s41467-024-46911-7)
Supplement: Supplementary file 3 — Description of Additional Supplementary Files [file 41467_2024_46911_MOESM3_ESM.pdf]

Description of Additional Supplementary Files

File Name: Supplementary Movie 1

Description: Demonstration of two packaging-free m-$HSbP_2O_8$-MXene MSCs connected in series to power a calculator under repeated bending.
